# Evaluation of a validated methylation triage signature for human papillomavirus positive women in the HPV FOCAL cervical cancer screening trial

**DOI:** 10.1002/ijc.31976

**Published:** 2018-12-24

**Authors:** Darrel A. Cook, Mel Krajden, Adam R. Brentnall, Lovedeep Gondara, Tracy Chan, Jennifer H. Law, Laurie W. Smith, Dirk J. van Niekerk, Gina S. Ogilvie, Andrew J. Coldman, Rhian Warman, Caroline Reuter, Jack Cuzick, Attila T. Lorincz

**Affiliations:** ^1^ BC Centre for Disease Control Vancouver British Columbia Canada; ^2^ BC Cancer Agency Vancouver British Columbia Canada; ^3^ Lower Mainland Pathology and Laboratory Medicine Vancouver British Columbia Canada; ^4^ Faculty of Medicine University of British Columbia Vancouver British Columbia Canada; ^5^ Wolfson Institute of Preventive Medicine, Centre for Cancer Prevention, Queen Mary University of London London United Kingdom

**Keywords:** human papillomavirus, methylation, cervical cancer screening, colposcopy triage, cervical cancer

## Abstract

Human papillomavirus (HPV)‐based cervical cancer screening requires triage of HPV positive women to identify those at risk of cervical intraepithelial neoplasia grade 2 (CIN2) or worse. We conducted a blinded case–control study within the HPV FOCAL randomized cervical cancer screening trial of women aged 25–65 to examine whether baseline methylation testing using the S5 classifier provided triage performance similar to an algorithm relying on cytology and HPV genotyping. Groups were randomly selected from women with known HPV/cytology results and pathology outcomes. Group 1: 104 HPV positive (HPV+), abnormal cytology (54 CIN2/3; 50 <CIN2); Group 2: 103 HPV+, normal cytology with HPV persistence at 12 mo. (53 CIN2/3; 50 <CIN2); Group 3: 50 HPV+, normal cytology with HPV clearance at 12 mo. (assumed <CIN2), total n=257. For the combined groups, S5 risk score CIN2/3 relative sensitivity, specificity and positive predictive value (PPV) were compared with other triage approaches. Methylation showed a highly significant increasing trend with disease severity. For CIN3, S5 relative sensitivity and specificity were: 93.2% (95%CI: 81.4–98.0) and 41.8% (35.2–48.8), compared to 86.4% (75.0–95.7) and 49.8% (43.1–56.6) respectively for combined abnormal cytology/HPV16/18 positivity (differences not statistically significant at 5% level); adjusted PPVs were 18.2% (16.2–20.4) and 19.3% (16.6–22.2) respectively. S5 was also positive in baseline specimens from eight cancers detected during or after trial participation. The S5 methylation score had high sensitivity and PPV for CIN3, compatible with US and European thresholds for colposcopy referral. Methylation signatures can identify most HPV positive women at increased risk of cervical cancer from their baseline screening specimens.

## Abstract 2

**What's new?**

DNA methylation testing could simplify the triage process for screening HPV+ women for cervical cancer, according to new results from a case‐control study. Most pre‐cancerous cervical lesions do not progress to cancer, so triage is done to identify those lesions more likely to become cancerous and boost screening specificity. Here, the authors tested women in the HPV FOCAL study for baseline methylation using the S5 classifier. Methylation signatures, they found, performed with 93% sensitivity and 18% PPV for CIN3, comparable to the combination of cytology and HPV genotyping (86% sensitivity and 19% PPV).

**Abbreviations**
HPV: human papillomavirus
PPV: positive predictive value
CIN: cervical intraepithelial neoplasia
LBC: liquid‐based cytology
NILM: negative for intraepithelial lesions and malignancy
RCT: randomized controlled trial
HC2: hybrid capture 2 high‐risk HPV test
ASCUS: atypical squamous cells, undetermined significance
STM: specimen transport medium
ROC: receiver operating characteristic
RLU: relative light units
AUC: area under the ROC curve

## Introduction

Persistent high‐risk human papillomavirus (HPV) infection is the primary cause of cervical cancer.^1^, ^2^, ^3^ HPV‐based cervical screening can identify >95% of pre‐cancerous cervical lesions (cervical intraepithelial neoplasia [CIN] grade 2 or worse [CIN2+]),^4^ but has a relatively low specificity for CIN2+ because most HPV positive women have transient infections which spontaneously clear,^5^ with few progressing to CIN3 and cancer.^4^

Widespread adoption of primary HPV cervical screening has supported the search for a triage test which retains high sensitivity but increases specificity and positive predictive value (PPV), while accurately identifying women at high risk for CIN3+. Reflex liquid‐based cytology (LBC) is commonly used,^6^ but its low sensitivity (~50–70%)^7^ for CIN2+ limits its triage utility. Consequently, follow‐up is usually required to monitor for HPV clearance or persistence in women with no intraepithelial lesions or malignancy (NILM) LBC diagnoses. Triage strategies can also include HPV16 and HPV18 (HPV16/18) genotyping together with LBC.^8^, ^9^ Immediate colposcopy referral is recommended in some countries for HPV16/18 positive women regardless of cytology diagnosis, and also for women with other HPV types who have abnormal LBC. HPV positive, LBC negative women are subsequently re‐tested to identify persistent HPV infections with referral of these to colposcopy.^8^ Another triage strategy is p16/Ki67 immunostaining which is more sensitive than standard LBC and identifies women at elevated risk of CIN2+,^10^ but the interpretation still requires subjective microscopy. An objective triage strategy which could be automated and incorporated as a reflex molecular test following HPV screening would be advantageous. DNA methylation assays targeting host and/or HPV genes may meet this requirement as they have been shown to have higher sensitivity and similar specificity to LBC for identifying CIN2+.^11^, ^12^, ^13^

The S5 DNA methylation classifier was developed in a London UK colposcopy referral population^14^ and was later validated with cervical screening samples.^15^ It is based on targeting late regions of HPV16, HPV18, HPV31 and HPV33 combined with the promoter region of the human tumour suppressor gene *EPB41L3*. HPV FOr CerviCAL Cancer Screening (HPV FOCAL) is a population‐based Canadian randomized controlled trial (RCT) comparing HPV versus LBC for primary cervical cancer screening.^16^, ^17^ The trial provided an ideal study for additional validation of “real‐world” molecular triage test performance. We assess the S5 methylation classifier for detecting histopathologically confirmed CIN2/3 vs. <CIN2 among HPV positive HPV FOCAL trial women.

## Materials and Methods

### HPV FOCAL Trial Design

The HPV FOCAL RCT^16^, ^17^, ^18^ (ISRCTN79347302) compared HPV (Hybrid Capture® 2 High‐Risk HPV DNA Test® [HC2]; Qiagen Inc., Germantown, MD, USA) (Intervention and Safety [HPV] Arms) versus LBC screening (Control Arm) in women aged 25–65. HC2 positive (HC2+) women in the HPV Arms were triaged by LBC, with immediate colposcopy referral for abnormal cytological findings. Women with NILM cytology were re‐screened 12 months later, with those who remained HC2+ and/or had abnormal cytology referred to colposcopy (Supporting Information Fig. S1). HPV genotyping was included in the trial as an adjunct study,^19^ which allowed modeling the performance of combination triage approaches using both cytology and HPV16/18 genotyping. Women were randomly enrolled into one of the three FOCAL Trial arms until closure of the Safety Arm, after which randomization continued to the Intervention and Control arms (final enrollment: Intervention Arm, 9552; Control, 9457; Safety, 6214). Round 1 screening, follow‐up and management were identical for the two HPV Arms, so these were combined for the present analysis. After excluding 22 women with invalid/incomplete baseline HC2 results, the HPV Arms included 15,744 women. Colposcopy examination included biopsy and/or endocervical curettage. CIN diagnoses were based on histopathology. Written informed consent was obtained from all trial participants. Both the RCT (H06‐04032) and the nested methylation case–control study (H14‐02974) were approved by the University of British Columbia/BC Cancer Agency Clinical Research Ethics Board.

### Methylation Case–Control Study Population

We focused on baseline HPV positive women detected by the HC2 test. Women were classified into three groups based on their HC2 and reflex LBC results (Table 1). Group 1: HC2+, LBC ≥atypical squamous cells of undetermined significance (ASCUS; referred to colposcopy at baseline); Group 2: HC2+, LBC NILM at baseline, remained HC2+ and/or had LBC ≥ASCUS at the 12‐month subsequent screen (referred to colposcopy at 12 months); Group 3: HC2+, LBC NILM at baseline with HPV clearance at 12 months (not referred to colposcopy; assumed to have <CIN2 histopathology). At enrollment, a duplicate cervical sample collected in specimen transport medium (STM; Qiagen) was stored at −80°C for molecular studies. For groups 1 and 2, STM samples were randomly selected from all women with CIN2/3 and <CIN2 in each group to achieve approximately equal distribution of CIN2/3 and <CIN2. For group 3, STM samples were randomly selected from all samples meeting the group definition. The three groups were combined to estimate methylation test characteristics for HC2+ triage. In addition, samples were tested from eight women from any study arm who developed invasive cervical cancer during or after the trial; these women with malignancy were not included in Groups 1–3, nor the sensitivity, specificity, PPV or receiver operating characteristic (ROC) calculations. Personal identifying information was removed and a unique ID number was applied to each study sample prior to methylation analyses.

**Table 1:** HPV FOCAL S5 methylation case–control study design

Description	Number in case–control study		Number in HPV FOCAL population (Intervention & Safety Arms combined)	
Baseline HC2+ (all)			1290 (8.2%)	
Group 1 HC2+/LBC ≥ASCUS (“HPV prevalent/abnormal cytology group”; all CIN2/3 in this group were identified at the baseline screen)	104		481 (466 attended colposcopy)	
	54 CIN2/3	50 <CIN2	150 CIN2+ (32.2%)	316 <CIN2 (67.8%)
LBC NILM^a^			809 (753 attended 12 mo. subsequent screen)	
Group 2 HC2+ and/or LBC ≥ASCUS at 12 mo. subsequent screen (“HPV persistence group”; all CIN2/3 in this group were identified at the 12 mo. subsequent screen)	103		422 (56%) (403 attended colposcopy)	
	53 CIN2/3	50 <CIN2	92 CIN2+ (22.8%)	311 <CIN2 (77.2%)
Group 3 HC2 negative and LBC NILM at 12 mo. subsequent screen (“HPV clearance group”) Colposcopy was not performed after subsequent screen; this group was assumed to be <CIN2^b^.	50		331 (44.0%)	
		50 <CIN2		
Baseline HC2 negative (all)			14,454	
Total	257		15,744	

HC2: hybrid capture 2 high‐risk HPV test; LBC: liquid‐based cytology; ASCUS: atypical squamous cells, undetermined significance; NILM: negative for intraepithelial lesions and malignancy; CIN: cervical intraepithelial neoplasia. CIN2+: includes CIN2, CIN3 and invasive cancer.

a Groups 2 and 3 were selected from this trial subset.

b Passive follow‐up through the screening registry of women in this group revealed five women who were subsequently referred to colposcopy in the seven to nine years after baseline with no CIN2+ lesions detected.

### Sample Preparation, HPV Genotyping, and Methylation Testing

HPV16/18 genotyping was done by the cobas® 4800 HPV test (Roche Molecular Systems, Pleasanton CA). The Linear Array HPV Genotyping Test (Roche) was used to genotype cobas “other high risk positive” specimens. A HPV16/18 genotype was assigned if the specimen was cobas positive for one or both of HPV16 or HPV18, regardless of the detection of any other HPV type(s).

DNA was extracted from 200μL of each STM sample (MagMAX™ Total Nucleic Acid Isolation Kit; Life Technologies, Burlington ON, Canada), eluted into 40μL, and used for methylation testing. DNA concentrations were estimated and DNA was shipped on dry ice to the Wolfson Institute laboratory where methylation testing was done as previously described.^14^ Lab personnel were blinded to the sample group assignment, HPV genotype and CIN outcomes.

### Statistical Analysis

All analyses were based on a pre‐specified statistical analysis plan. The main hypothesis was that S5 methylation triage at baseline had equivalent sensitivity and PPV to triage by baseline LBC ≥ASCUS or LBC NILM and HPV16/18 positivity (LBC ≥ASCUS/HPV16/18). Histopathologically confirmed CIN2/3 versus <CIN2 was used as the reference standard.

The S5 risk score is based on methylation levels of the human gene *EPB41L3* together with HPV16L1, HPV16L2, HPV18L2, HPV31L1 and HPV33L2. The PCR‐based assay was followed by quantitative pyrosequencing to measure methylation levels of each assay component. The S5 risk score was calculated as: S5 = 30.9(*EPB41L3*) + 13.7(HPV16L1) + 4.3(HPV16L2) + 8.4(HPV18L2) + 22.4(HPV31L1) + 20.3(HPV33L2); a score of ≥0.8 indicated a positive methylation test.^14^ For a full listing of the anonymized methylation line data versus CIN endpoints please contact the corresponding author.

HC2 relative light unit (RLU)/cutoff ratios, where a positive test was ≥1.0, were used as a surrogate for HPV viral load; a higher ratio indicated higher viral load.

Relative sensitivity and specificity (i.e., relative to the FOCAL Trial triage for HC2+ women as described in the trial design) for cumulative round 1 CIN2/3 and CIN3 during the trial were calculated for S5 performed at baseline; 95% non‐parametric bootstrapped CIs were obtained from 10,000 bootstrap replicates. Unadjusted PPVs were calculated by dividing the number of women with CIN2/3 or CIN3 cervical lesions (true positive screens) by the number with a positive triage test in the methylation study subset.^17^ PPVs were also adjusted for CIN2/3 and CIN3 prevalence estimates (26.8% and 12.2% respectively) for the trial HPV arms (Table 1), using the following formula: PPV = (Sn*Pr)/((Sn*Pr)+(1−Sp)*(1−Pr)), where Sn is sensitivity, Sp is specificity, and Pr is the CIN2/3 or CIN3 prevalence. To place S5 triage in context, the same parameters were calculated at baseline for triage by: 1) LBC ≥ASCUS/HPV16/18 (the main comparison); 2) HPV16/18 positive; and 3) LBC ≥ASCUS. The S5 colposcopy referral rate was estimated using the S5 positive rates for CIN2/3 versus <CIN2 in the case–control study, and extrapolating to the distribution of CIN2/3 and <CIN2 for all HC2+ women in the HPV arms of the trial by re‐weighting the sampling groups according to the trial population data (Table 1). Colposcopy referral rates for the other three triage strategies were calculated from round 1 trial data for the HPV arms. Wilson's method was used to calculate 95%CI. Cuzick's test^20^ was used to test for trend in S5 scores by disease category (ordered <CIN2, CIN2, CIN3 and cancer) and by HPV viral load. McNemar's test was used to explore differences in paired nominal data.

S5 ROC curves were generated for CIN2/3 and CIN3 by re‐weighting the sampled groups as described above, from which area under the ROC curve (AUC) with 95%CI was calculated from a non‐parametric empirical bootstrap. The combined ROC estimated the classification performance of S5 and its components for all HC2+ women in the HPV arms of the trial.

Statistical calculations were performed using R version 3.3.1.

## Results

Relative to the HPV FOCAL triage algorithm (Supporting Information Fig. S1), which was used as the reference standard, the S5 classifier had sensitivities for CIN2/3 and CIN3 of 75.7% (95%CI: 67.3–83.7) and 93.2% (95%CI: 84.8–100.0) respectively (Table 2). S5 sensitivity was significantly greater than either cytology or HPV16/18 genotyping (Table 2) but was not significantly different (CIN2/3: *p*=0.170; CIN3: *p*=0.248) than the sensitivity of combination triage by LBC ≥ASCUS/HPV16/18. S5 relative specificities for <CIN2 and <CIN3 [44.0% (95%CI: 36.1–52.2) and 41.8% (95%CI: 35.3–48.4) respectively] were similar to LBC ≥ASCUS/HPV16/18 triage, but were lower than both LBC ≥ASCUS and HPV16/18 triage (Table 2). The adjusted PPVs of S5 for CIN2/3 (33.1%) and CIN3 (18.2%) were similar to the corresponding PPVs for triage by LBC ≥ASCUS/HPV16/18 (34.2% for CIN2/3 and 19.3% for CIN3), and for LBC ≥ASCUS alone (35.7% and 19.1% respectively), but lower than for HPV16/18 triage alone (44.4% and 28.1% respectively).

**Table 2:** First screening round cumulative CIN2/3 and CIN3 relative sensitivity, specificity and positive predictive value of the S5 methylation classifier and other triage approaches.

Triage approach	Colposcopy referral rate^a^ (95%CI)	CIN2/3						CIN3					
		n	Relative sensitivity (95%CI)	McNemar *p* value^b^	Relative specificity (95%CI)	Unadjusted PPV (95%CI)	Adjusted PPV^c^ (95%CI)	n	Relative sensitivity (95%CI)	McNemar *p* value^b^	Relative specificity (95%CI)	Unadjusted PPV (95%CI)	Adjusted PPV^c^ (95%CI)
S5 positive at baseline	4.3 (4.0–4.6)	81	75.7% (67.3–83.7)		44.0% (36.1–52.2)	49.1% (41.5–59.9)	33.1% (29.3–37.3)	41	93.2% (84.8–100.0)		41.8% (35.3–48.4)	24.8% (18.3–31.5)	18.2% (16.2–20.4)
LBC ≥ASCUS or LBC NILM and HPV16/18 positive at baseline^d^	4.2 (3.9–4.5)	73	68.2% (59.3–77.0)	0.170	52.0% (43.9–60.0)	50.3% (42.4–58.5)	34.2% (29.7–39.3)	38	86.4% (75.0–95.7)	0.248	49.8% (43.1–56.6)	26.2% (18.9–33.3)	19.3% (16.6–22.2)
cobas HPV16/18 positive at baseline^d^	2.1 (1.9–2.3)	53	49.5% (40.2–59.1)	<0.001	77.3% (70.3–83.9)	60.9% (50.6–70.9)	44.4% (36.3–54.1)	32	72.7% (59.5–86.0)	0.008	74.2% (68.2–80.0)	36.8% (27.0–47.0)	28.1% (22.6–34.7)
LBC ≥ASCUS at baseline	3.0 (2.8–3.3)	54	50.5% (41.1–59.8)	<0.001	66.7% (59.1–74.3)	51.9% (42.2–61.9)	35.7% (29.4–43.0)	27	61.4% (46.7–75.6)	0.001	63.8% (57.5–70.4)	26.0% (17.7–34.6)	19.1 % (14.7–24.0)
HPV FOCAL Round 1^e^ (LBC ≥ASCUS at baseline OR LBC NILM at baseline and HC2+ at 12 mo. follow‐up)	5.9 (5.5–6.3)	107	Ref	<0.001	Ref	27.9% (25.2–30.9)		44	Ref	0.248	Ref	12.7% (10.7–15.0)	

The individual tests and combinations were modeled on baseline screening results, except for the HPV FOCAL Round 1 approach and those based on HPV genotyping, which also considered the 12‐month subsequent specimen results. CIN: cervical intraepithelial neoplasia; CI: confidence interval; PPV: positive predictive value; HC2: hybrid capture 2 HPV test; cobas: cobas® 4800 HPV test; LBC: liquid‐based cytology; ASCUS: atypical squamous cells, undetermined significance; NILM: negative for intraepithelial lesions and malignancy; Ref: reference; the sensitivity and specificity of each modeled triage approach are relative to those for the FOCAL trial.

a The colposcopy referral rate for S5+ triage was estimated for the overall trial HPV arms by re‐weighting the case–control study subset data. Rates for the other triage approaches were based on trial data for the HPV arms. Rates are per 100 women screened.

b McNemar *p* value for each triage strategy compared to S5. We performed an additional McNemar's test for S5 vs. LBC≥ASCUS/HPV16/18 for CIN3, where the S5 cutoff was adjusted to 0.91 so that the specificities of the two triage approaches were the same (*p* = 0.617).

c Due to oversampling of women with CIN2/3 lesions in the methylation study subset, adjusted PPVs were also calculated using the CIN2/3 and CIN3 prevalence for the FOCAL trial HPV arms: adjusted PPV = (Sn*Pr)/((Sn*Pr)+(1−Sp)*(1−Pr)), where Sn is sensitivity, Sp is specificity, and Pr is the CIN2/3 or CIN3 prevalence.

d Baseline LBC NILM/HPV16/18 positive women were only referred to colposcopy if they were HC2 positive and/or LBC≥ASCUS at the 12 month subsequent test. Thus, we are unable to determine how many LBC NILM women may have had CIN2/3 at baseline, and were HC2 negative/LBC NILM at the 12 month test.

e Published trial colposcopy referral rate and PPVs obtained from Ogilvie et al. 2016^17^. The number of CIN2/3 and CIN3 cases is the total for the methylation case–control study subset.

The estimated colposcopy referral rate for S5 methylation classifier positive women (4.3%) was higher than for HPV16/18 positive and LBC ≥ASCUS triage, but was similar to the combined strategy of LBC ≥ASCUS/HPV16/18 triage (4.2%), which was our most sensitive and main comparison. The highest referral rate was for the full FOCAL trial triage approach (5.9%) which detected all 107 CIN2+ cases.

Of the 107 CIN2/3 cases, 81 (76%) were S5 positive at baseline. FOCAL triage identified 54 (50%) at baseline and the remaining 53 (50%) cases after 12 month re‐screening. For the 44 CIN3 cases, 41 (93%) were S5 positive at baseline. FOCAL triage identified 27 (61%) at baseline and the remaining 17 (39%) cases at 12 months (Table 3).

**Table 3:** High‐grade CIN detected by S5 vs. FOCAL trial triage at baseline and 12 month subsequent screens

	CIN2/3 (n = 107)		CIN3 (n = 44)	
	Detected at baseline screen	Detected after 12 mo. subsequent screen	Detected at baseline screen	Detected after 12 mo. subsequent screen
S5 triage	81 (76%)	n/a	41 (93%)	n/a
FOCAL trial triage	54 (50%)	53 (50%)	27 (61%)	17 (39%)

CIN, cervical intraepithelial neoplasia; n/a, not applicable

**Figure 1:**
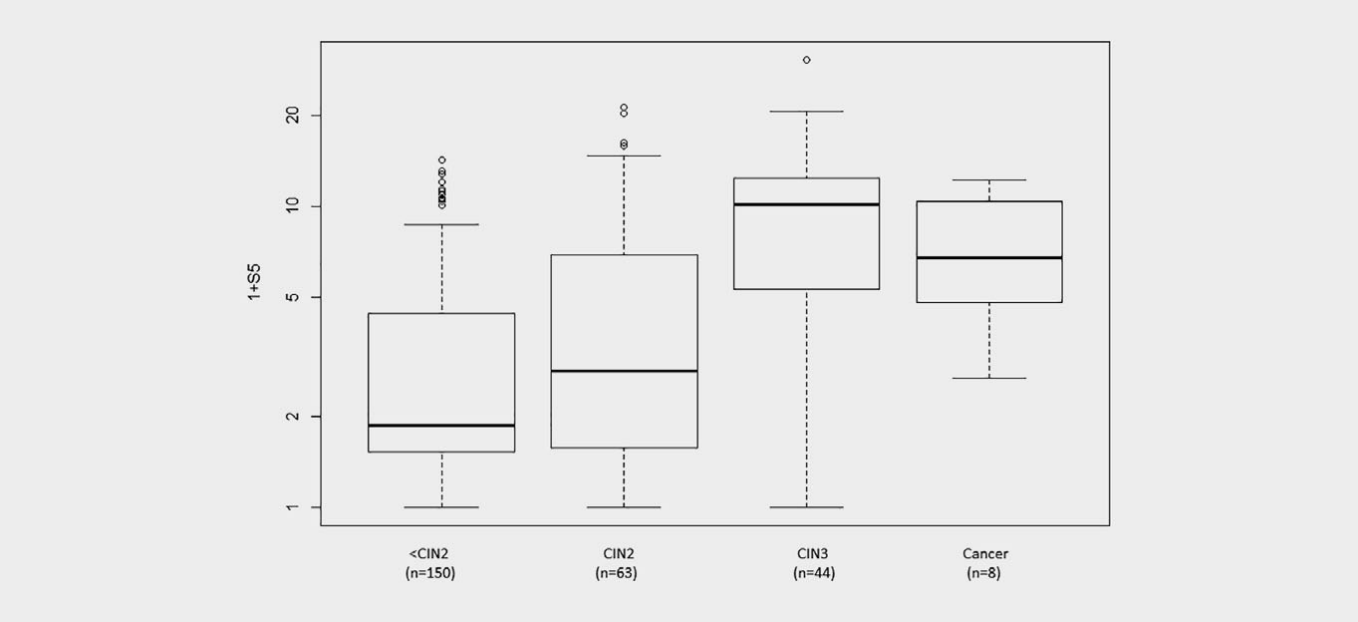
S5 score distributions by CIN diagnosis. CIN, cervical intraepithelial neoplasia. Note: The middle line is the median; the box shows the inter‐quartile range (IQR) and the whiskers extend to at most 1.5 times the IQR. Cancer S5 scores include only those for the baseline samples taken between 4 and 67 months before cancer diagnosis.

Figure 1 illustrates the median S5 scores, stratified by CIN diagnosis, for women in groups 1–3 combined and those diagnosed with cervical cancers. Median S5 scores showed a significantly increasing trend with both lesion severity (Supporting Information Table 1; Cuzick *p*_trend_<0.0001) and with HPV viral load (Supporting Information Table 2; *p*_trend_=0.0001). Women with <CIN2 and LBC ≥ASCUS, LBC NILM or non‐HPV16 positivity had median scores near the S5 cutoff, while HPV16 positive women had a higher median S5 score, similar to some of the women with high‐grade disease and cancer (Supporting Information Table 1).

**Figure 2:**
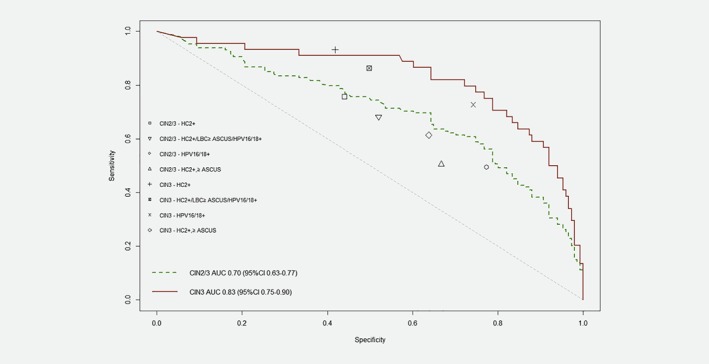
S5 receiver operating characteristic curves, CIN2/3 and CIN3. HC2: hybrid capture 2 HPV test; CIN: cervical intraepithelial neoplasia; ASCUS: atypical squamous cells, undetermined significance; AUC: area under the curve; CI: confidence interval. The markings shown in the legend illustrate CIN2/3 and CIN3 point estimates for HC2+ women, and for each modeled triage option. [Color figure can be viewed at wileyonlinelibrary.com]

S5 ROC curves for CIN2/3 and CIN3 are shown in Figure 2; for CIN2/3 the AUC was 0.70 (95%CI: 0.64–0.77) and for CIN3 was 0.83 (95%CI: 0.75–0.90). Figure 2 also shows CIN2/3 and CIN3 ROC point estimates for women based on HPV genotype and reflex cytology triage combinations.

All baseline specimens from the eight invasive cervical cancer cases were S5 positive and all cases were HPV16 or HPV18 positive on the baseline or 12‐month subsequent‐to‐baseline sample (Table 4). Of these cancers, six were adenocarcinomas and two were squamous cell carcinomas. For the four individuals who had another sample post‐baseline but prior to diagnosis of the cancer, the S5 scores had increased. All trial‐detected cancers underwent secondary review by a senior trial pathologist and all were confirmed to be of cervical origin.

**Table 4:** S5 scores, HPV genotype(s), HPV viral load and LBC results for women who developed invasive cervical cancers

Study Arm	Baseline sample				Subsequent sample			Cancer type	Age at cancer diagnosis
	Months prior to cancer diagnosis	HPV Type^a^/LBC Result	S5 Score	Viral load^b^	Months prior to cancer diagnosis	HPV Type(s)^a^/LBC Result	S5 Score		
CA	67	HPV16/NILM	5.8	404.25				Squamous large cell non‐keratinizing	62
SA	4	HPV18/ASCH	11.3	65.47				Adenocarcinoma	41
CA	25	NT/NILM	5.0	16.31	1	HPV18/ ATYENNEO	9.4	Adenocarcinoma	48
CA	6	HPV18/LSIL	1.7	2.20				Adenocarcinoma	35
CA	40	NT/NILM	2.9	0.64	6	HPV18/ASCH	12.4	Adenocarcinoma	33
CA	34	NT/NILM	5.8	124.91	5	HPV16, HPV84/ ATYEMET	18.5	Adenocarcinoma	44
SA	7	HPV16/NILM	10.6	125.78				Adenocarcinoma	28
CA	30	NT/NILM	7.3	0.20	2	HPV18/HSIL	27.0	Squamous large cell non‐keratinizing	50

LBC, liquid‐based cytology; CA, control arm; SA, safety arm; NT, not tested; NILM, negative for intraepithelial lesions and malignancy; ASCH, atypical squamous cells, cannot rule out high‐grade; ATYENNEO, atypical endocervical cells, favour neoplastic; LSIL, low‐grade squamous intraepithelial lesion; ATYEMET, atypical endometrial cells; HSIL, high‐grade squamous intraepithelial lesion

a HPV16 and 18 were identified by the cobas 4800 HPV test; other HPV types were identified by the Linear Array HPV Genotyping test.

b Hybrid capture 2 relative light unit/cutoff ratios were used as a surrogate for HPV viral load, where the threshold for a positive test was ≥1.0; higher ratios indicate a higher viral load.

Details of the S5 negative CIN2 and CIN3 cases are shown in Supporting Information Table 3. For CIN3, one case was HPV58 positive and another was both HPV52 and HPV68 positive; these are HPV types not included in the S5 classifier. The third CIN3 case was associated with HPV67 which was detected only in the 12‐month subsequent‐to‐baseline specimen. HPV67 has been designated as possibly carcinogenic to humans,^21^ but is not included in most commercial high‐risk HPV screening assays. For CIN2, most S5 negative cases were also associated with HPV types not included in the S5 classifier, but one S5 negative CIN2 case was HPV18 positive at baseline, another was HPV33 positive and two additional cases had HPV16 detected only in the 12‐month subsequent‐to‐baseline specimens.

## Discussion

We observed a moderate baseline sensitivity of the S5 DNA methylation classifier for CIN2 and a high sensitivity (>90%) for CIN3 and cancer among HPV positive women. S5 specificities were lower but PPVs were comparable to other accepted triage methods. Compared to the FOCAL trial triage of colposcopy referral for HPV positive women with baseline abnormal reflex cytology or NILM baseline cytology with 12‐month HPV persistence, methylation triage can provide objective and more timely identification of most women with high‐grade cervical lesions at baseline screening. Of women with CIN3, S5 detected 93% of cases at baseline, compared to 61% for the FOCAL trial baseline triage. For CIN2/3 the percentages were 76% for S5 triage and 50% for FOCAL triage, respectively.

S5 methylation testing had similar triage performance for detection of CIN2/3 at baseline compared to a triage approach based on immediate colposcopy referral for women with LBC ≥ASCUS, or LBC NILM with HPV16/18 positivity, a triage approach used predominantly in the US.^22^ Our trial did not include an option for colposcopy referral of baseline LBC NILM, HPV16/18 positive women, as this was not recommended in Canada when the FOCAL trial was designed. In addition, baseline LBC NILM, HPV16/18 positive women would not have been referred to colposcopy unless the 12 month subsequent specimen was HC2 positive or LBC ≥ASCUS. Thus, we were not able to determine how many additional CIN2/3 would have been detected among baseline HPV 16/18 positive women in the trial by the US approach. However, that approach would have increased colposcopy referral rates, which goes against our search for triage strategies that can reduce over‐treatment.^23^ In the HPV arms of the trial, S5 triage would have reduced clinician visits and screen tests as more high‐grade disease would have been detected at baseline, thus simplifying the screening algorithm and potentially reducing loss to follow‐up. In future, methylation markers may be shown to preferentially detect advanced lesions with a high short term risk of cervical cancer; indeed, a recent study from the POBASCAM trial showed that women negative for DNA methylation had a low future risk of cervical cancer over the subsequent 14 years.^24^

An earlier study of S5^15^ among women in the Predictors 3 (P3) trial, whose initial screen was cytology with subsequent HPV testing, reported S5 CIN2+ and CIN3+ sensitivities of 74% and 84% respectively for HPV positive women, similar to our study (75.7% and 93.2% respectively). However, <CIN2 and <CIN3 specificities for S5 in the P3 study (65% and 63%) vs. FOCAL (44.0% and 41.8%) were higher. The lower S5 specificity in our study may partly be related to the relatively high S5 scores obtained for HPV16 positive women with <CIN2. Furthermore, women in the FOCAL HPV arms underwent HPV primary screening rather than cytology. It seems plausible that primary cytology screening may preferentially detect later stage disease because HPV screening detects more transient HPV infections in addition to the persistent HPV infections responsible for CIN2+, and thus, S5 triage might be expected to have lower specificity among women screened for HPV. A review of studies of host gene methylation in cervical cancer^13^ revealed wide methylation variations in the same gene between different studies, some of which may be related to population differences and/or the methylation testing methodology.

Performance characteristics for methylation studies (not including those with self‐collected samples) using a variety of genes^25^ reported CIN2+ sensitivities ranging from 48%‐89% in populations initially screened by either HPV or cytology, and 44%‐90% in colposcopy referral populations. Specificities ranged from 50%‐81% and 49%‐95% respectively. The S5 sensitivity in the FOCAL case–control study is consistent with the upper range of results of these studies, whereas specificity is within the lower range. Of note, the areas under the ROC curve for FOCAL (CIN2/3: 0.70; CIN3: 0.83) are consistent with other studies of both screening (CIN2+ 0.72−0.80; CIN3+ 0.84) and colposcopy referral (CIN2+ 0.82; CIN3+ 0.77–0.97) populations.^25^

Sensitivity and specificity was not reported for the FOCAL trial as there was no verification performed for negative screens. We used the FOCAL triage approach as the reference method; thus, the sensitivities for other single and combination triage approaches reported in this paper are relative to those based on the FOCAL trial, which were assumed for comparison purposes to be ~100%. The relative CIN3 sensitivity for the S5 classifier (93.2%) was similar to FOCAL while that for CIN2/3 was lower (75.7%). This might be expected given that most of the S5 negative CIN2+ cases were associated with non‐HPV16/18/31/33 genotypes. Targeting additional HPV genotypes in the S5 classifier might improve sensitivity, but could result in lower specificity. Methylation triage including the *EPB41L3* or other host genes has been reported to have comparable performance to cytology for HPV positive women,^26^ although cytology performed slightly better, especially when attempting to maximize the sensitivity of methylation triage.^25^ S5 methylation triage has also been shown to be more sensitive for CIN2+ than HPV16/18 genotyping and displayed similar specificity.^15^ Triage based on HPV16/18 positivity in our study (CIN2/3 sensitivity: 49.5%%; CIN3: 72.7%) compared to S5 (CIN2/3 sensitivity: 75.7%; CIN3: 93.2%) is consistent with this observation.

Of eight women who developed cervical cancers during or after FOCAL trial participation, two were HC2 negative on the baseline specimen. All eight cancers were S5 positive at the baseline screen, but the median S5 score for the baseline samples for women with cancers was lower than for women with CIN3 (5.8 vs. 9.3). Some of the tested samples from subjects with cancer were obtained several years prior to the cancer diagnosis, which could have resulted in lower S5 scores than if samples had been tested closer to their cancer diagnoses. This is likely the case, as the four women who had a subsequent sample tested had substantially higher S5 scores than for their baseline samples. Moreover, six of the cancers tested were adenocarcinomas and it has been reported that these tend to display lower methylation levels compared to squamous cell carcinomas.^25^, ^27^

At least two methylation assays based on human genes are commercially available for HPV positive triage. The GynTect® assay is based on ASTN1, DLX1, ITGA4, RXFP3, SOX17 and ZNF671,^28^ while the QIAsure Methylation Test Kit is based on promoter hypermethylation of FAM19A4 and hsa‐mir‐124‐2.^29^ The S5 classifier utilizes the *EPB41L3* human gene, which was found to have the best performance in an earlier credentialing study of a number of human genes in the Predictors 1 and 2 studies.^30^ S5 triage sensitivity for CIN3 was higher than for the GynTect® assay (93% vs. 65%) but GynTect® had higher specificity (42% vs. 89%).^31^ Using two types of self‐collected samples tested by the same methylation components as the QIAsure assay, De Strooper et al.^32^ reported CIN3+ sensitivities of 68%‐71% and specificities of 68%‐76%. Sensitivity improved to 85%‐89%, but specificity was lower at 46%‐55%, when methylation was combined with HPV16/18 genotyping. Further research will be needed to optimize the sensitivity and specificity of methylation assays for triage.

A strength of our study is that the samples were obtained from a RCT embedded within an organized cervical screening program, with high compliance to colposcopy recommendations, standardized colposcopic examinations with biopsy, and centralized blinded pathology review. An important limitation of our case–control study is that it was retrospective because the trial was not designed specifically to assess prospectively additional molecular triage methods in HPV positive women. In addition, although women with CIN2/3 and <CIN2 were randomly selected from the population of women meeting those criteria, it is possible that the methylation‐tested sub‐population is not representative of all women in the trial with CIN2/3 and <CIN2. Optimal ethnic and geographically representative validation of S5 triage will require additional studies designed to directly compare S5 with established strategies, preferably with colposcopy referral for all women with a positive triage test. An intriguing question is whether S5 classifier negative CIN2+ reflects lesions destined to regress spontaneously, or result from the S5 classifier not including targets for some high‐risk genotypes. To understand this phenomenon would require systematic follow up of CIN2+ women who are undergoing assessment for CIN progression or regression.

In conclusion, DNA methylation assessed by the S5 classifier correlates strongly with aggressive cervical disease, showing high sensitivity for CIN3 and cancer, the raison d’être for a cervical screening program. S5 PPV for CIN3 is compatible with both US and European colposcopy referral thresholds.^33^, ^34^ Methylation tests have the potential to simplify triage by more quickly identifying HPV‐infected women in need of colposcopy. Of the 107 CIN2/3 in our follow‐up study, 81 cases were identified at baseline by S5 as compared to 73 by combination LBC ≥ASCUS/HPV16/18 triage; the remaining 34 women were diagnosed only after 12 months of follow‐up. Thus, S5 can detect a greater proportion of high‐grade disease with a high short‐term risk of cervical cancer at the baseline screen than the other approaches, which can lessen concerns of losing women during follow‐up.

## Authors' Contributions

Study conception and design: M. Krajden, A. Lorincz, D. Cook, A. Brentnall. Acquisition, analysis and interpretation of data: D. Cook, A. Brentnall, L. Smith, L. Gondara, M. Krajden, A. Lorincz, D. van Niekerk, G. Ogilvie, A. Coldman. Writing, review, and revision of the manuscript: D. Cook, A. Lorincz, A. Brentnall, M. Krajden, J. Cuzick. Laboratory analyses: J. Law, T. Chan, R. Warman, C. Reuter. All authors reviewed the manuscript for critical intellectual content and agreed to submission of the manuscript for publication.

## Supporting information


**Supplementary Figure 1. HPV FOCAL Trial Schematic**
The yellow highlighted area illustrates the trial subset used for the methylation case‐control study.

**Supplementary Table 1.** Median baseline S5 scores by reflex cytology grade and HPV genotype
**Supplementary Table 2.** Median baseline S5 scores by HPV viral load
**Supplementary Table 3.** HPV, cytology and viral load results for S5 negative CIN2+ cases
