# Supplementary material for: Evaluation of a validated methylation triage signature for human papillomavirus positive women in the HPV FOCAL cervical cancer screening trial
Source: Int J Cancer. 2018 Dec 24;144(10):2587–95. doi: 10.1002/ijc.31976 (PMC6492122; doi:10.1002/ijc.31976)
Supplement: Supplementary file 1 — Supplementary Figure 1. HPV FOCAL Trial Schematic The yellow highlighted area illustrates the trial subset used for the methylation case‐control study. [file IJC-144-2587-s001.docx]

Supplementary Figure 1. HPV FOCAL Trial schematic


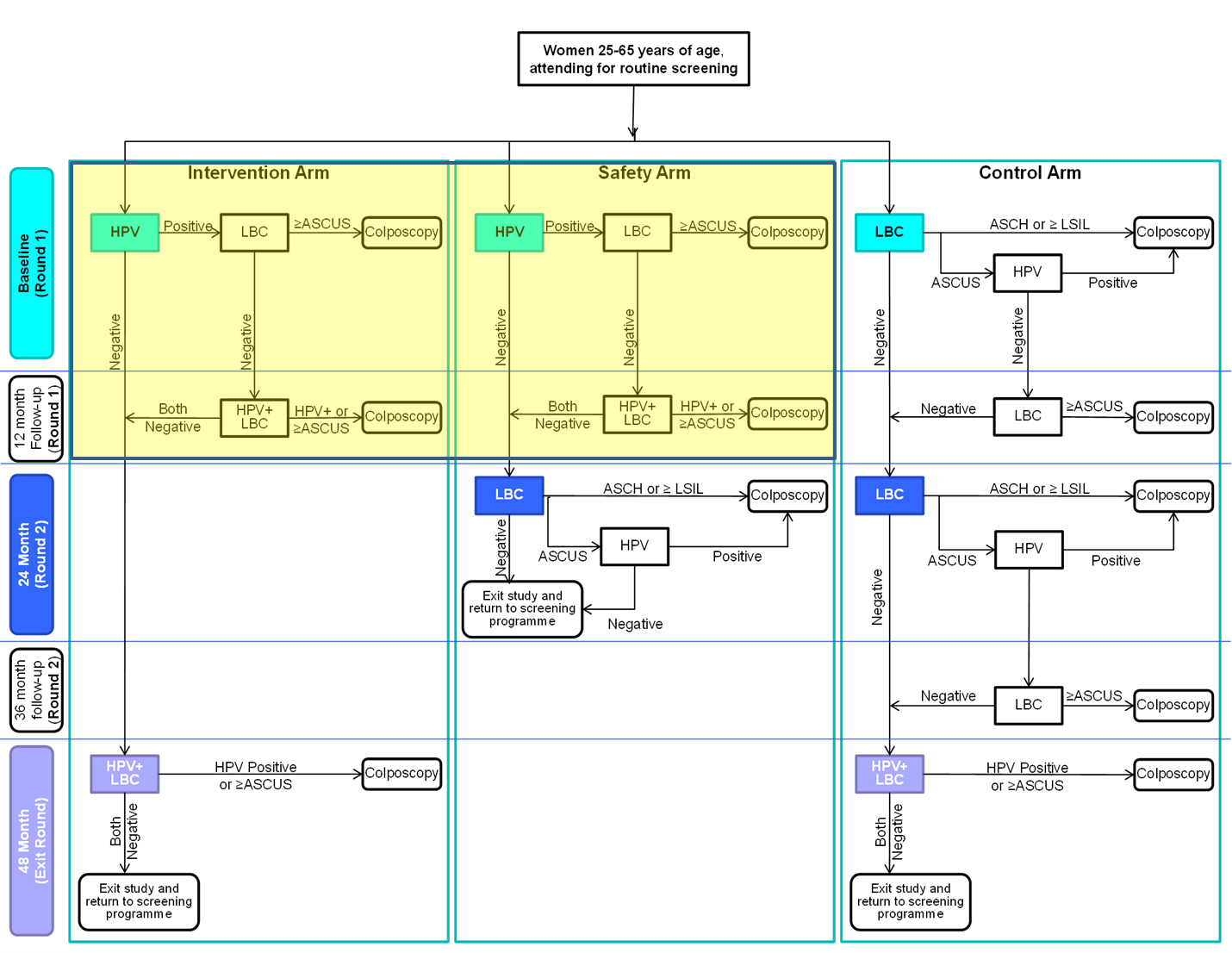


The yellow highlighted area illustrates the trial subset used for the methylation case-control study.

Refs: Ogilvie G, van Niekerk D, Krajden M, et al. Effect of screening with primary cervical HPV testing vs cytology testing on high-grade cervical intraepithelial neoplasia at 48 months: The HPV FOCAL randomized clinical trial. JAMA 2018;320(1):43-52; Ogilvie GS, Krajden M, van Niekerk D, et al. HPV for cervical cancer screening (HPV FOCAL): Complete Round 1 results of a randomized trial comparing HPV-based primary screening to liquid-based cytology for cervical cancer. Int J Cancer. 2017;140(2):440-448.
