# Supplementary material for: Evaluation of a validated methylation triage signature for human papillomavirus positive women in the HPV FOCAL cervical cancer screening trial
Source: Int J Cancer. 2018 Dec 24;144(10):2587–95. doi: 10.1002/ijc.31976 (PMC6492122; doi:10.1002/ijc.31976)
Supplement: Supplementary file 2 — Supplementary Table 1. Median baseline S5 scores by reflex cytology grade and HPV genotype Supplementary Table 2. Median baseline S5 scores by HPV viral load Supplementary Table 3. HPV, cytology and viral load results for S5 negative CIN2+ cases [file IJC-144-2587-s002.docx]

Supplementary Table 1. Median baseline S5 scores by reflex cytology grade and HPV genotype

|  | Median S5 score (inter-quartile range) | | | | p_trend_ |
| --- | --- | --- | --- | --- | --- |
|  | <CIN2  n=150 | CIN2  n=63 | CIN3  n=44 | Cancers  n=8 |  |
| LBC ≥ASCUS  n=104 | 0.9  (0.3-4.8) | 1.5  (0.7-8.1) | 9.6  (4.9-11.2) | 6.5  (^a^) | <0.0001 |
| LBC NILM  n=153 | 0.9  (0.6-2.8) | 2.2  (0.4-5.1) | 8.0  (1.1-13.0) | 5.8  (4.5-8.1) | <0.0001 |
| cobas HPV16+^b^  n=71 | 6.3  (3.2-9.3) | 8.7  (5.9-14.4) | 10.3  (7.1-12.6) | 8.2  (^a^) | 0.0007 |
| cobas non-HPV16+^b^  n=149 | 0.8  (0.5-2.4) | 0.8  (0.4-2.7) | 1.5  (0.9-7.9) | 6.5  (^a^) | 0.015 |
| All  n=257 | 0.9  (0.5-3.4) | 1.8  (0.6-6.1) | 9.2  (4.1-11.5) | 5.8  (3.4-9.8) | <0.0001 |

CIN: cervical intraepithelial neoplasia; LBC: liquid-based cytology; ASCUS: atypical squamous cells, undetermined significance; NILM: negative for intraepithelial lesions and malignancy; cobas: Roche cobas® 4800 HPV test

^a^2 observations

^b^HPV16+ includes all HPV16 positive samples regardless of the detection of any other high-risk HPV type(s) in a given sample; non-HPV16+ includes those HPV16 negative, but positive for any other high-risk HPV type(s)

Supplementary Table 2. Median baseline S5 scores by HPV viral load

|  | Viral load^a^ | | | | p_trend_ |
| --- | --- | --- | --- | --- | --- |
|  | <10  n=90 | 10 to 99.9  n=74 | 100 to 499.9  n=55 | ≥500  n=38 |  |
| Median S5 score (inter-quartile range) | 0.8  (0.6-3.0) | 1.5  (0.6-6.3) | 3.4  (0.7-7.6) | 5.1  (0.8-8.6) | 0.0001 |

^a^Hybrid capture 2 relative light unit/cutoff ratios were used as a surrogate for HPV viral load, where the threshold for a positive test was ≥1.0; higher ratios indicate a higher viral load.

Supplementary Table 3. HPV, cytology and viral load results for S5 negative CIN2+ cases

| Diagnosis | Case # | Baseline Specimen | | | | | 12 Month Subsequent Specimen | | | |
| --- | --- | --- | --- | --- | --- | --- | --- | --- | --- | --- |
|  |  | Viral load^a^ | cobas HPV Result | Linear Array HPV Genotypes | LBC Result | S5 Score | Viral load^a^ | cobas HPV Result | Linear Array HPV Genotypes | LBC Result |
| CIN3 | 1 | 155.1 | non-HPV16/18 positive | HPV58 | NILM | 0.2 | 104.5 | NT | NT |  |
|  | 2 | 1202.7 | non-HPV16/18 positive | HPV52, HPV68 | NILM | 0.5 | 2081.3 | non-HPV16/18 positive | NT |  |
|  | 3 | 2.8 | HPV negative | HPV negative | NILM | 0.0 | 3.2 | HPV negative | HPV67 |  |
| CIN2 | 4 | 282.4 | HPV negative | HPV56 | ASCUS | 0.7 |  |  |  |  |
|  | 5 | 96.34 | non-HPV16/18 positive | HPV52 | ASCUS | 0.4 |  |  |  |  |
|  | 6 | 44.5 | HPV 18 and non-HPV16/18 positive | HPV18, HPV40, HPV51 | LSIL | 0.7 |  |  |  |  |
|  | 7 | 2776.6 | non-HPV16/18 positive | HPV42, HPV51 | HSIL | 0.4 |  |  |  |  |
|  | 8 | 20.9 | non-HPV16/18 positive | HPV45, HPV83 | LSIL | 0.5 |  |  |  |  |
|  | 9 | 1423.7 | non-HPV16/18 positive | HPV51, HPV58, HPV62 | LSIL | 0.79 |  |  |  |  |
|  | 10 | 23.2 | non-HPV16/18 positive | HPV59 | NILM | 0.2 | 2087.5 | non-HPV16/18 positive | HPV35 |  |
|  | 11 | 110.9 | non-HPV16/18 positive | HPV51, HPV62 | NILM | 0.3 | 600.6 | non-HPV16/18 positive | HPV51, HPV53, HPV62 |  |
|  | 12 | 2.3 | non-HPV16/18 positive | HP6, HPV52 | NILM | 0.0 | 1427.5 | non-HPV16/18 positive | HPV31, HPV52, HPV54 |  |
|  | 13 | 7.4 | non-HPV16/18 positive | HPV52 | NILM | 0.2 | 54.6 | non-HPV16/18 positive | HPV52 |  |
|  | 14 | 7.3 | non-HPV16/18 positive | HPV56 | NILM | 0.6 | 1.8 | non-HPV16/18 positive | HPV negative |  |
|  | 15 | 4.3 | HPV negative | HPV66 | NILM | 0.78 | 1372.8 | non-HPV16/18 positive | HPV33 |  |
|  | 16 | 155.7 | non-HPV16/18 positive | HPV35 | NILM | 0.0 | 31.7 | HPV16 and non-HPV16/18 positive | HPV16, HPV31, HPV54, HPV56, HPV70 |  |
|  | 17 | 8.5 | HPV negative | NT | NILM | 0.4 | 5.7 | HPV negative | HPV52, HPV62 |  |
|  | 18 | 27.9 | non-HPV16/18 positive | HPV45, HPV51 | NILM | 0.6 | 47.0 | non-HPV16/18 positive | HPV45, HPV51 |  |
|  | 19 | 164.4 | non-HPV16/18 positive | HPV56, HPV59, CP6108 | NILM | 0.4 | 131.0 | non-HPV16/18 positive | HPV56, HPV58, HPV66 |  |
|  | 20 | 7.9 | non-HPV16/18 positive | HPV52 | LSIL | 0.77 |  |  |  |  |
|  | 21 | 28.7 | non-HPV16/18 positive | HPV66 | NILM | 0.4 | 6.1 | non-HPV16/18 positive | HPV39, HPV66 |  |
|  | 22 | 431.1 | non-HPV16/18 positive | HPV56, HPV62 | ASCH | 0.4 |  |  |  |  |
|  | 23 | 17.5 | non-HPV16/18 positive | HPV35, HPV58, HPV84 | HSIL | 0.2 |  |  |  |  |
|  | 24 | 209.2 | non-HPV16/18 positive | HPV33, HPV58 | NILM | 0.5 | 64.1 | non-HPV16/18 positive | HPV58 |  |
|  | 25 | 14.1 | non-HPV16/18 positive | HPV45, HPV56 | NILM | 0.2 | 17.1 | HPV16 and non-HPV16/18 positive | HPV16, HPV31, HPV45, HPV84 |  |
|  | 26 | 30.0 | non-HPV16/18 positive | HPV39 | NILM | 0.7 | 33.6 | non-HPV16/18 positive | HPV39 |  |

Abbreviations: cobas: Roche cobas® 4800 HPV test; LBC: liquid-based cytology; CIN: cervical intraepithelial neoplasia; NILM: negative for intraepithelial lesions and malignancy; NT: not tested; ASCUS: atypical squamous cells, uncertain significance; LSIL: low-grade squamous intraepithelial lesion; HSIL: high-grade squamous intraepithelial lesion; ASCH: atypical squamous cells, cannot rule out high-grade;

^a^Hybrid capture 2 relative light unit/cutoff ratios were used as a surrogate for HPV viral load, where the threshold for a positive test was ≥1.0; higher ratios indicate a higher viral load.
